# The Influence of Cross-Fostering on Alcohol Consumption and Depressive-Like Behaviors in HA and LA Mice: The Role of the Endogenous Opioid System

**DOI:** 10.3390/brainsci11050622

**Published:** 2021-05-13

**Authors:** Agata Nawrocka, Piotr Poznański, Marzena Łazarczyk, Michał Gorzałczyński, Dominik Skiba, Renata Wolińska, Magdalena Bujalska-Zadrożny, Kabirullah Lutfy, Bogdan Sadowski, Mariusz Sacharczuk

**Affiliations:** 1 Department of Experimental Genomics, Institute of Genetics and Animal Biotechnology, Polish Academy of Sciences, 05-552 Magdalenka, Poland; a.nawrocka@igbzpan.pl (A.N.); m.lazarczyk@igbzpan.pl (M.Ł.); d.skiba@igbzpan.pl (D.S.); m.sacharczuk@igbzpan.pl (M.S.); 2 Department of Pharmacodynamics, Centre for Preclinical Research and Technology, Medical University of Warsaw, 02-091 Warsaw, Poland; m.gorzalczynski94@gmail.com (M.G.); renata.wolinska@wum.edu.pl (R.W.); magdalena.bujalska@wum.edu.pl (M.B.-Z.); 3 Department of Pharmaceutical Sciences, College of Pharmacy, Western University of Health Sciences, Pomona, CA 91766, USA; klutfy@westernu.edu; 4 Warsaw School of Engineering and Health, 02-366 Warsaw, Poland; sadbogd@yahoo.com

**Keywords:** ethanol dependence, depression, the opioid system, stress-induced analgesia, cross-fostering

## Abstract

The development of alcohol dependence and depression is determined by various genetic and environmental factors. In the presented study, we used high analgesia (HA) and low analgesia (LA) mouse lines, characterized by different endogenous opioid system activity and divergent blood–brain barrier permeability, to determine the influence of cross-fostering of these lines raised by surrogate mothers on ethanol consumption and development of depressive-like behaviors. We also investigated ethanol drinking by biological parents or surrogate mothers. Furthermore, we investigated whether these parental changes would alter the effect of naloxone on ethanol intake and depressive-like behaviors in offspring. Our results reveal that cross-fostering of HA and LA raised by surrogate mothers has a greater impact on depressive-like behaviors than ethanol consumption. Ethanol intake by biological parents substantially affected depressive-like behaviors and ethanol consumption in offspring. Moreover, ethanol intake by biological parents or an adoptive mother modified the effect of naloxone on ethanol consumption and preference and depressive-like behaviors in the HA offspring only. Together, these results indicate that cross-fostering differentially affects the effect of naloxone on alcohol consumption and the development of depression.

## 1. Introduction

Alcohol dependence represents a serious public health problem worldwide. Major public health and social hazards of alcoholism include cognitive decline [1], weakened social and familial connections [2], rise in crime and related issues or social status, losses, and a decrease in professional productivity [3]. Alcohol addiction is a very complex disorder, and several mechanisms have been implicated in its development. Generally, chronic alcohol consumption is maintained by positive and negative reinforcement, during which the subject consumes alcohol to experience pleasure or to avoid adverse effects of withdrawal, respectively. It seems that environmental incentives are important factors in the initiation and continuation of alcohol consumption.

The genetic background of subjects has a crucial role in the transition from occasional use to chronic intake and addiction [4]. A very powerful environmental stimulus that prompts alcohol use and abuse is chronic stress related to the childhood period (e.g., early separation from a mother) or social maturation (e.g., difficulties in interpersonal relationships) [5]. As documented in animal models, stressful stimuli differentially affect individuals and are dependent on age. Male rats separated immediately after weaning overuse ethanol compared with mature counterparts exposed to the same type of stimuli [6]. Social separation increases ethanol intake and preference in mice as well [7]. Other societal factors, such as forced endurance of a crowded place or domination/submission-like interactions between individuals in a group, elicit ethanol overuse [7]. A hostile societal environment or individual inherent deficits place subjects at risk of social relationship deprivation and generate motor overactivity and anxiety or depression in rodents [8]. These changes are likely mediated by serotonergic signaling decline or alterations in the functioning of the mesolimbic dopaminergic pathway [9].

Opioid peptides and opioid receptors are found in brain regions implicated in aversion, reward, stress, and addiction, suggesting an inevitable role of the endogenous opioid system in alcohol dependence [10]. In mice with decreased activity of the endogenous opioid system, a 5-fold increase in ethanol intake was observed under stressful conditions compared with the same strain housed in a neutral environment [11]. On the contrary, chronic stress had no marked influence on ethanol consumption in mice with a higher activity of this system. Therefore, it is suggested that prolonged stress uncovers a hidden but existing phenotype that contributes to ethanol addiction under a low activity of the opioid system [11].

The alcoholism–depression comorbidity is well-documented. However, the link between the two remains unclear and requires further research. Depressive symptoms in alcohol users may be induced by alcohol per se or may occur independent of drug use. Both addiction and mental disorders may be evoked by chronic stress and genetic factors, such as a congenitally reduced endogenous opioid system activity [12]. Depression is usually associated with chronic pain, likely originated from a dysfunctional opioid system. Treatment with some antidepressant drugs restores mood balance and alleviates pain [13].

The relationship between susceptibility to alcohol addiction, depression, and the level of opioid system functioning emerges from opioids’ involvement in nociception and mood-balancing or in support of survival by the induction of attraction to reward or aversion avoidance in response to external stimuli. The endogenous opioid system is altered by environmental factors starting at a very early stage of life [14]. The nucleus accumbens and ventral tegmental area are less susceptible to stress in the early postnatal phase, while the endogenous opioids in the amygdala are more reactive [15]. In rodent pups neglected by their parents, the early loss of biological caregivers or separation from a mother brings about long-lasting changes in endogenous opioid levels in brain areas associated with reward as well as changes in responsiveness to opioid receptor agonists or antagonists [15].

As postulated previously, endogenous opioid peptides play a key role in social bonding between parents and their offspring. It has been documented that mu opioid receptors (MOR) mediate a positive affective state activated by a mother [16]. Preclinical studies developed toward both opioid-mediated beneficial sensations following closeness with parents and experiencing adverse effects due to separation from them, similar to alcohol withdrawal, confirmed MOR involvement in arising pleasant sensation as mice with MOR inactivity appeared less intact under identical conditions [16]. Exposure to stress at a very early phase of life is a predictor of alcohol addiction in adults [17]. Multiple studies on non-human primates and rodents demonstrated stress-induced neurohormonal changes of the hypothalamic-pituitary-adrenal axis, morphological malformations in the brain, and alterations in the expression of the genes associated with the mesolimbic dopaminergic pathway [17].

In the current study, we used the mouse model of distinct response to stress, obtained by a selection toward high (HA) and low analgesia (LA) induced by forced swimming, referred to as HA/LA model, reviewed elsewhere [18]. These mouse lines exhibit differences in endogenous opioid system activity, exhibiting different ethanol consumption levels. However, these lines cannot be perceived as ethanol-preferring lines because both lines normally consume ethanol in an amount not exceeding 50% of their total daily fluids intake; therefore, the observed effect is exclusively pharmacological [11]. Our previous studies using HA and LA mouse lines proved that chronic mild stress (CMS) leads to depressive-like behaviors and increased nociception only in HA mice. Moreover, we showed that CMS stimulates ethanol drinking only in LA but not HA mice. When they consumed a small amount of ethanol, stress-induced depression and pain sensation declined [12].

The present study utilized the cross-fostering paradigm (CAP) to assess the influence of mother transposition on ethanol intake and preference and depressive-like behaviors in pups of the same or opposite line assigned as the surrogate. Additionally, we investigated the influence of ethanol intake by biological parents or surrogate mothers on susceptibility to ethanol intake and depressive-like behaviors in the offspring, along with assessing the impact of genetic and environmental factors related to parental rearing on the effect of naloxone on these parameters in the progeny.

## 2. Materials and Methods

### 2.1. Animal Model

All experiments were conducted on the 91st and 92nd generation of outbred Swiss-Webster mice of both sexes belonging to two divergently selected mouse lines. The selection criterion was a high (HA) and low (LA) level of swim stress-induced analgesia. Both mouse lines were maintained in the animal facility of the Institute of Genetics and Animal Biotechnology PAS. The selection procedure relied on forcing animals to swim for 3 min in a water pool at a temperature of 20 °C [19]. After swimming, mice were laid down on cellulose wadding and left to dry. Measurements of analgesia and nociception were performed by recording the latency to respond to a thermal stimulus (a hot plate at 56 °C) before swimming and after. Individuals characterized by the longest (55–60 s) and the shortest (up to 10 s) latency after swimming were chosen as progenitors of the next generation of HA and LA mice, respectively.

Mice of both lines were housed in polycarbonate cages (225 × 167 × 140 mm, Animalab, Poland) in groups of 4–5 mice of the same sex and closely related to each other with free access to water and food (LABOFEED H, Polska). Animals were housed in a room at constant temperature (22 ± 2 °C), air humidity (55 ± 5%), and an artificial 12 h light/dark cycle (light phase started at 7:00 a.m.). Experiments were performed upon approval of the II Local Ethics Committee on Animal Testing (consent no. WAW2/71/2016).

### 2.2. Experiment Design

**Figure 1:**
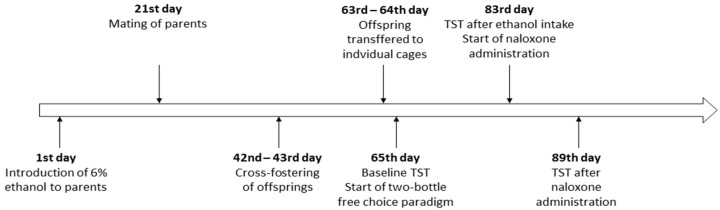
Timeline of the experimental procedure.

The experiment was divided into three phases (Figure 1). Parents of both lines were allowed to gain distinct background associated with ethanol use before mating. Parental generation of HA and LA lines were given access to only 6% ethanol solution for three weeks, during which ethanol intake was not measured (1st–21st day). On the other hand, ethanol-naïve parents had access only to water (1st–21st day). In the next step, HA and LA mice were mated within the same line to produce HA and LA offspring. The couples of a given strain were arranged as follows: male x female (both consuming only water), male x female (both having access only to ethanol), male with free access to ethanol x female receiving only water, and vice versa. Males were separated from females 18 days after mating, a standard procedure for breeding HA/LA lines. To avoid the possibility of fetal alcohol syndrome (FAS) development during pregnancy or nursing, females had no access to ethanol. There were no withdrawal symptoms observed (e.g., hyperactivity, aggressive behavior) in any mice (21st day).

In the second phase, on the day of parturition, newborn mouse pups delivered by LA or HA mothers were transferred to a Petri dish wadded with sawdust taken from a cage of the surrogate mother (42nd–43rd day). Five min later, a neonate was placed in a box with a surrogate. The offspring exchange was performed between or within HA and LA mothers of similar labor time (± 6–8 h) and litter quantity. After three weeks, infants were weaned from surrogates and transferred to individual cages. Ultimately, 8 study groups per each of the two lines were obtained (Table 1). Four to six mice were included in each group (Table 2). To control the litter effect, we used offspring from two different litters. Due to rare cases of a mother giving birth to nine females and only two males, groups of parental variant 8 HA consisted of two males and two females when pups were transferred to a surrogate of the opposing line. As the control group, we used non-transferred pups raised by their biological parents, which did not drink ethanol. Then ethanol intake was measured in a two-bottle free-choice paradigm, where mice could freely choose between two 50 mL bottles, one with tap water and one with a 6% ethanol solution. The amount of the given solution consumed was measured every three days with an accuracy of 0.01 g. The positioning of bottles was changed daily to avoid any side-preference. During this step, bottles were also checked for spillage. In the next step, ethanol preference was calculated. Surprisingly, we did not observe any differences between ethanol intake or preference among females and males (in HA variant 8 with transfer to a surrogate of opposed line).

During the last phase, naloxone was administered intraperitoneally to each mouse for six consecutive days, starting on the 83rd day of the experiment, and its effect on ethanol intake was assessed. Over that period, ethanol intake and preference were measured as before.

### 2.3. Drugs

Naloxone (NLX)-4,5-epoksy-3,14-dihydroksy-17-(prop-2-enylo)-morfinian-6-on is a synthetic derivative of oxymorphone. As a full and non-selective opioid receptors antagonist, the drug displays affinity to all three opioid receptors; however, the highest affinity was documented for µ compared with δ and κ receptors. Naloxone hydrochloride (TOCRIS, Bristol, Great Britain) was dissolved in 0.9% NaCl and then injected intraperitoneally to mice at a single dose of 27.5 µM/kg, which corresponds to a dose of 10 mg/kg, in a volume of 0.1 mL/10 g. The dose of NLX was chosen based on a previous study [20].

### 2.4. Assessment of Depressive-Like Behaviors

The development of depressive-like behaviors in mice after ethanol consumption and naloxone administration was assessed by the tail suspension test (TST) [21]. Measurements were performed at three time-points: before access to ethanol (65th day), before naloxone administration (83rd day), and after completion of naloxone treatment (89th day). For the TST, a wooden cage (680 × 365 × 280 mm) with the front removed was used. Each mouse was hung 120 mm from the box walls by its tail with adhesive tape to the fabric string glued to the box cover. Total immobility period (the duration of time that mouse paws remained still along with the head directed down) was calculated for a 6 min timeframe using the EthoVision system (Noldus, Wageningen, The Netherlands).

### 2.5. Statistics

Results were evaluated using Statistica 13.1 software (StatSoft, Inc., Tulsa, OK, USA) and are presented as mean values ± SE. Statistical significance was set at *p* ≤ 0.05. Normal distribution of data, assessed by D Agostin and Pearson tests, was examined with consideration of time, using two- or three-way analysis of variance (ANOVA). As an independent factor for ANOVA, we used the mouse line, treatment, and parental variant together. Parametric post hoc analysis was performed using the NIR test. The *p*-values of all significant comparisons are presented in Supplementary Materials (Tables S1–S12). Non-normally distributed data were analyzed by Mann–Whitney or Kruskal–Wallis test comparing two or more than two groups, respectively.

## 3. Results

### 3.1. Changes in Ethanol Intake and Preference in the HA and LA Mice Reared by Surrogates of the Same Line

**Figure 2:**
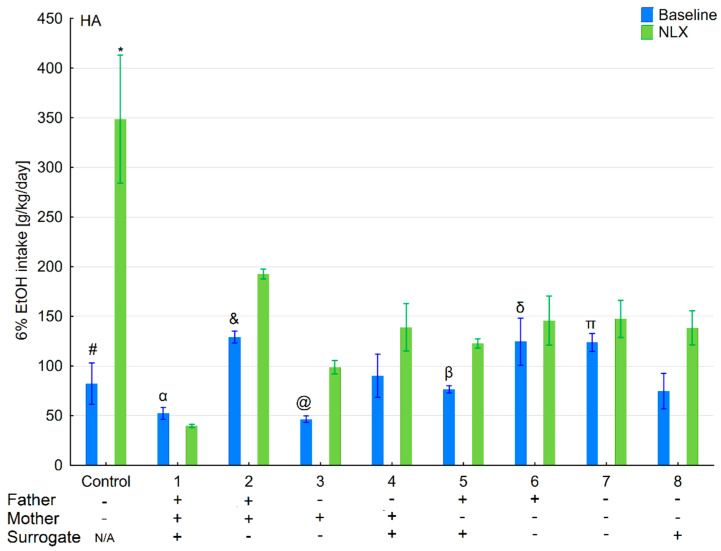
Effect of naloxone on 6% ethanol intake in the HA offspring reared by HA surrogate. The HA offspring reared by their biological mothers were used as controls. Post hoc comparisons are marked when *p*-value is at least less than 0.05: *, baseline vs NLX; #, control baseline vs. 2,6,7 baseline; α, 1 baseline vs. 2,4,6,7 baseline; &, 2 baseline vs. 3,5,8 baseline; @, 3 baseline vs. 4,6,7 baseline; β, 5 baseline vs. 6,7 baseline; δ, 6 baseline vs. 8 baseline; π, 7 baseline vs. 8 baseline.

**Figure 3:**
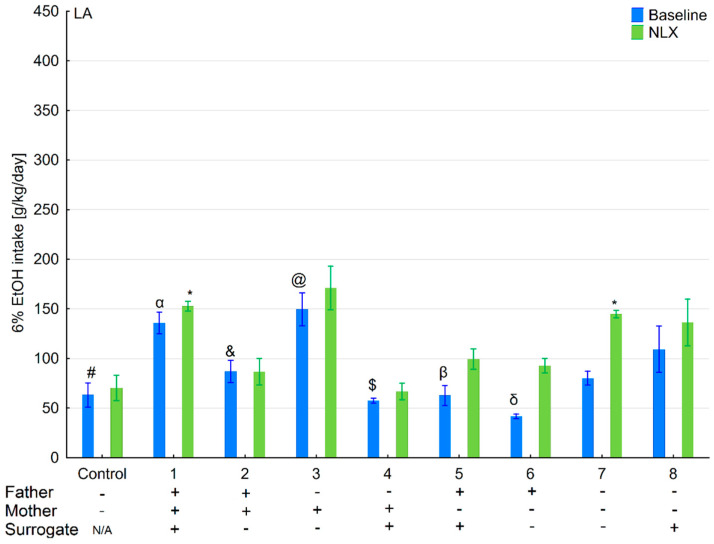
Effect of naloxone on 6% ethanol intake in the LA offspring reared by LA surrogate. The LA offspring reared by their biological mothers were used as controls. Post hoc comparisons are marked when *p*-value is at least less than 0.05: *, baseline vs. NLX; #, control baseline vs. 1,3,7,8 baseline; α, 1 baseline vs. 2,4,5,6,7 baseline; &, 2 baseline vs. 3,6,8; @, 3 baseline vs. 4,5,6,7; $, 4 baseline vs. 8 baseline; β, 5 baseline vs. 8 baseline; δ, 6 baseline vs. 7,8 baseline.

**Figure 4:**
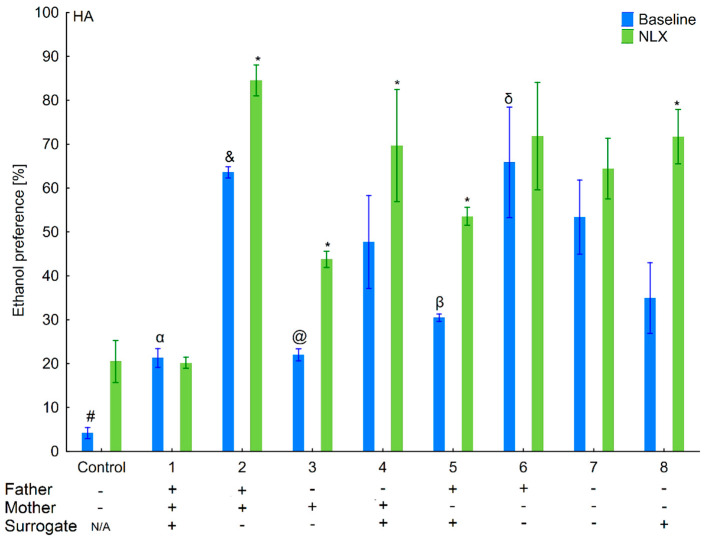
Effect of naloxone on 6% ethanol preference in the HA offspring reared by HA surrogate. The HA offspring reared by their biological mothers were used as controls. Post hoc comparisons are marked when *p*-value is at least less than 0.05: *, baseline vs NLX; #, control baseline vs. 2,4,5,6,7,8 baseline; α, 1 baseline vs. 2,4,6,7 baseline; &, 2 baseline vs. 3,5,8 baseline; @, 3 baseline vs. 4,6,7 baseline; β, 5 baseline vs. 6,7 baseline; δ, 6 baseline vs. 8 baseline.

**Figure 5:**
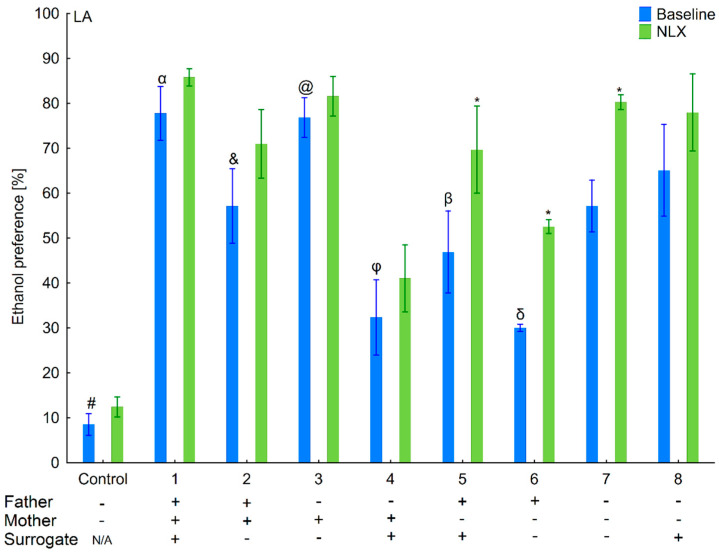
Effect of naloxone on 6% ethanol preference in the LA offspring reared by LA surrogate. The LA offspring reared by their biological mothers were used as controls. Post hoc comparisons are marked when *p*-value is at least less than 0.05: *, baseline vs. NLX; #, control baseline vs. 1,2,3,4,5,6,7,8 baseline; α, 1 baseline vs. 2,4,5,6,7 baseline; &, 2 baseline vs. 3,4,6; @, 3 baseline vs. 4,5,6,7 baseline; φ, 4 baseline vs. 7, 8 baseline; β, 5 baseline vs. 8 baseline; δ, 6 baseline vs. 7,8 baseline.

Ethanol intake and preference were measured in the two-bottle free-choice paradigm in HA and LA mice (Figure 2, Figure 3, Figure 4 and Figure 5). Two-way ANOVA taking line and variant as independent factors revealed that ethanol intake did not differ between lines (*F*(1,89) = 0.076; *p* = 0.78–line) or variants (*F*(8,89) = 1.65; *p* = 0.12–variant). However, LA mice expressed higher ethanol preference than HA ones (*F*(1,89) = 14.11; *p* < 0.001–line). Moreover, ethanol preference significantly differed between variants of adoption (*F*(8,89) = 10.75; *p* < 0.001–variant). Effects of adoption variant on ethanol intake and preference were more pronounced in the LA line as shown by a significant Line × Variant interaction (*F*(8,89) = 8.07; *p* < 0.001–ethanol intake; *F*(8,89) = 10.38; *p* < 0.001–ethanol preference).

Further analysis of the data using a one-way ANOVA (variant as an independent factor) within lines showed that variant of adoption had a significant impact on further ethanol intake (*F*(8,45) = 3.54; *p* < 0.01–HA line; *F*(8,44) = 7.27; *p* < 0.001–LA line) and ethanol preference (*F*(8,45) = 9.48; *p* < 0.001–HA line; *F*(8,44) = 11.63; *p* < 0.001–LA line).

### 3.2. Effects of Naloxone on Ethanol Intake and Preference in the HA and LA Mice Reared by Surrogates of the Same Line

Three-way ANOVA taking line, treatment, and variant as independent factors surprisingly indicated that administration of NLX did not affect ethanol intake (*F*(1,178) = 0.007; *p* = 0.93–treatment) (Figure 2, Figure 3, Figure 4 and Figure 5). Despite a non-significant effect of treatment, the interaction between the Line × Treatment was highly significant (*F*(1,178) = 20.96; *p* < 0.001), meaning that the effect of NLX administration on ethanol intake was more prominent in HA mice. Moreover, three-way ANOVA revealed that the treatment was more effective in some adoption variants (*F*(8,178) = 13.61; *p* < 0.001–Variant × Treatment interaction) considering their ethanol intake. A significant Line × Variant × Treatment interaction (*F*(8,178) = 10.48; *p* < 0.001) suggested that several adoption variants within the HA line were more sensitive to NLX treatment than the LA line concerning their ethanol intake.

Further two-way ANOVA (treatment and variant as independent factors) showed that NLX was effective in modifying ethanol intake in both HA (*F*(1,90) = 8.37; *p* < 0.01–treatment) and LA (*F*(1,88) = 14.71; *p* < 0.001–treatment) mice. In both lines, we observed that administration of NLX in several variants had higher effect on ethanol intake than in others as shown by a significant Variant × Treatment interaction (*F*(8,90) = 17.27, *p* < 0.001–HA line; *F*(8,88) = 2.09; *p* < 0.05–LA line).

Taking into consideration the ethanol preference, three-way ANOVA (line, treatment, and variant as independent factors) indicated that NLX treatment was effective (*F*(1,178) = 48.38, *p* < 0.001–treatment). Non-significant interactions showed that effect of NLX administration on ethanol preference was comparable between variants (*F*(8,178) = 0.94, *p* = 0.48–Variant × Treatment) and lines (*F*(1,178) = 0.83, *p* = 0.36–Line × Treatment). Additionally, a non-significant Line × Variant × Treatment interaction (*F*(8,178) = 1.14; *p* = 0.34) indicated that the effect of treatment on ethanol preference was identical among variants within each line.

Analysis within the lines (treatment and variant as independent factors) revealed that effect of NLX administration on ethanol preference was observed in both lines, but was more prominent in HA (*F*(1,90) = 28.59; *p* < 0.001–treatment) than LA (*F*(1,88) = 19.99; *p* < 0.001–treatment) mice. Adoption variants did not alter responsiveness to NLX treatment on ethanol preference as shown by a non-significant Variant × Treatment interaction (*F*(8,90) = 1.28; *p* = 0.26–HA line; *F*(8,88) = 0.76; *p* = 0.64–LA line).

### 3.3. Changes in the Level of Depressive-Like Behaviors in the HA and LA Mice Reared by a Surrogate of the Same Line

**Figure 6:**
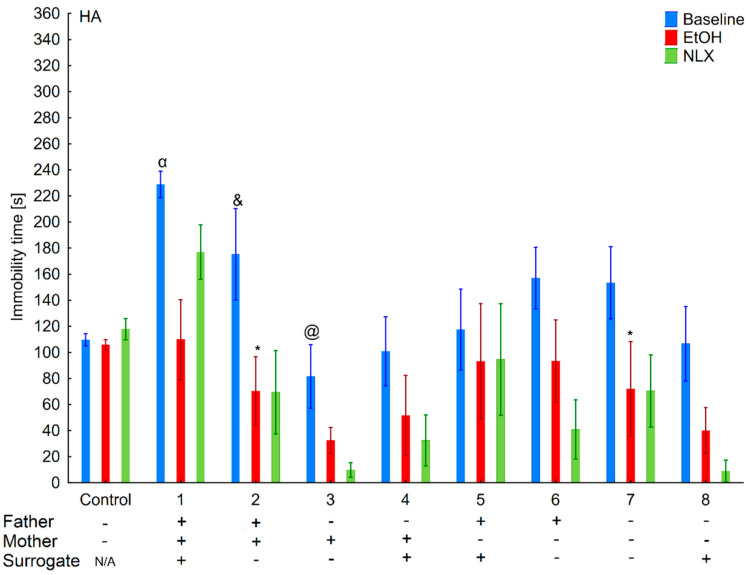
Effect of cross-fostering within the line, ethanol consumption, and NLX treatment on depressive-like behaviors of the HA individuals. The HA offspring reared by their biological mothers were used as controls. Post hoc comparisons are marked when *p*-value is at least less than 0.05: *, baseline vs. EtOH; α, 1 baseline vs. 3,4,5,7,8, and control baseline; &, 2 baseline vs. 3,4 baseline; @, 3 baseline vs. 6 baseline.

**Figure 7:**
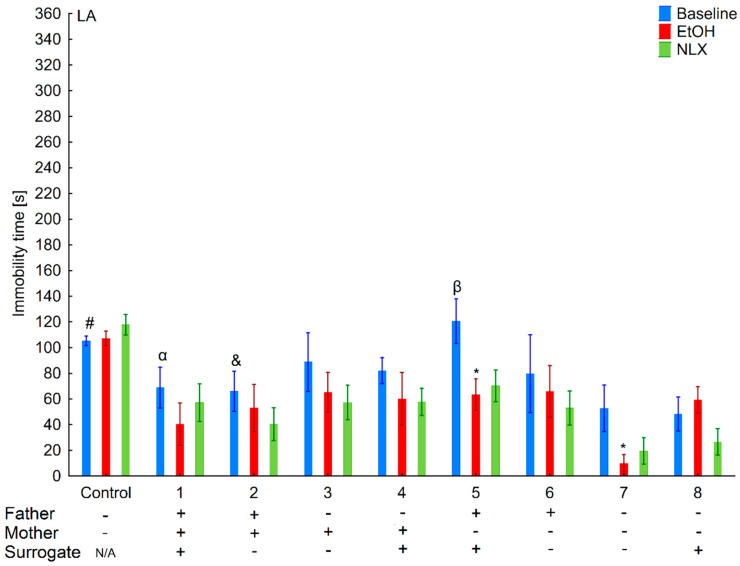
Effect of cross-fostering within the line, ethanol consumption, and NLX treatment on depressive-like behaviors of the LA individuals. The LA offspring reared by their biological mothers were used as controls. Post hoc comparisons are marked when *p*-value is at least less than 0.05: *, baseline vs. EtOH; #, control baseline vs. 7,8 baseline; α, 1 baseline vs. 5 baseline; &, 2 baseline vs. 5 baseline; β-5 baseline vs. 7,8 baseline.

Two-way ANOVA (line and variant as independent factors) showed that HA mice displayed longer immobility time than LA mice (*F*(1,86) = 28.35; *p* < 0.001–line) (Figure 6 and Figure 7). Moreover, depressive-like behaviors were more pronounced in mice from adoptions variants within the HA line as confirmed by a significant Line × Variant interaction (*F*(8,86) = 3.33; *p* < 0.01).

Analysis within lines (variant as independent factor) revealed that variants of adoption had a significant effect on this parameter only in the HA (*F*(8,43) = 3.13; *p* < 0.01) but not in LA (*F*(8,43) = 1.59; *p* = 0.15) mice.

### 3.4. Effects of Ethanol Consumption on Depressive-Like Behaviors in the HA and LA Mice Reared by a Surrogate of the Same Line

Three-way ANOVA (line, EtOH intake, and variant as independent factors) revealed that ethanol consumption had an anti-depressive effect (*F*(1,170) = 28.31; *p* < 0.001–EtOH intake) (Figure 6 and Figure 7). Moreover, the ethanol intake impact on depressive-like behaviors was more prominent in the HA than in LA mice, as shown by a significant Line × Treatment interaction (*F*(1,170) = 7.06; *p* < 0.01). A non-significant Treatment × Variant (*F*(8,170) = 0.74; *p* = 0.65) interaction and Line × Treatment × Variant (*F*(8,170) = 0.78; *p* = 0.62) showed that ethanol’s effect between adoption variants among lines was comparable.

Two-way ANOVA within the lines (EtOH intake and variant as independent factors) revealed that ethanol consumption reduced depressive-like behaviors more effectively in the HA (*F*(1,86) = 22.00; *p* < 0.001–EtOH intake) than LA (*F*(1,84) = 6.66; *p* < 0.05–EtOH intake) mice. A non-significant Variant × Treatment interaction (*F*(8,86) = 0.78; *p* = 0.78–HA line; *F*(8,84) = 0.77; *p* = 0.63–LA line) revealed that adoption variant did not alter the effect of ethanol intake on depressive-like behavior.

### 3.5. Effects of Naloxone on Depression-Like Behaviors in the LA and HA Mice Reared by a Surrogate of the Same Line

Three-way ANOVA (line, variant, NLX treatment as independent factors) indicated that NLX administration had no effect on depressive-like behaviors (*F*(1,168) = 0.30; *p* = 0.58–NLX factors), confirming that depressive-like behaviors remained the same as before the treatment (*F*(1,86) = 0.26; *p* = 0.69–HA line; *F*(1,82) = 0.18; *p* = 0.67–LA line) (Figure 5 and Figure 6).

### 3.6. Changes in Ethanol Intake and Preference in the HA and LA Mice Reared by Surrogates of the Opposite Line

**Figure 8:**
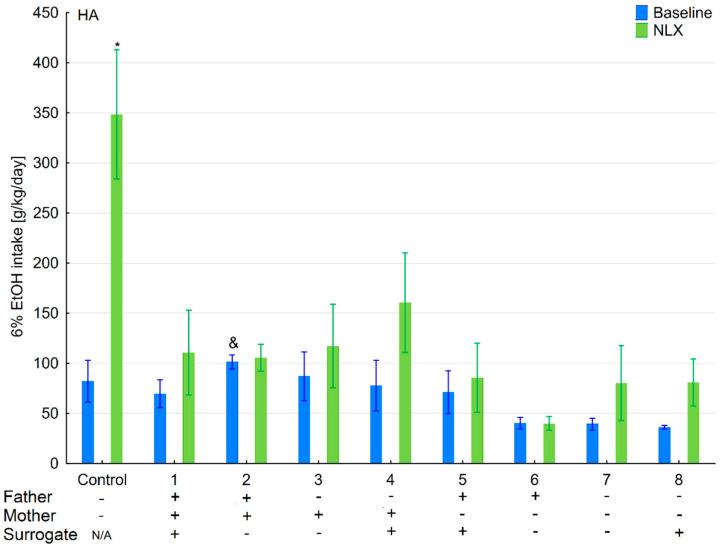
Effects of naloxone on 6% ethanol intake in the HA offspring reared by an LA surrogate. The HA offspring reared by their biological mothers were used as controls. Post hoc comparisons are marked when *p*-value is at least less than 0.05: *, baseline vs. NLX; &, 2 baseline vs. 6,7 baseline.

**Figure 9:**
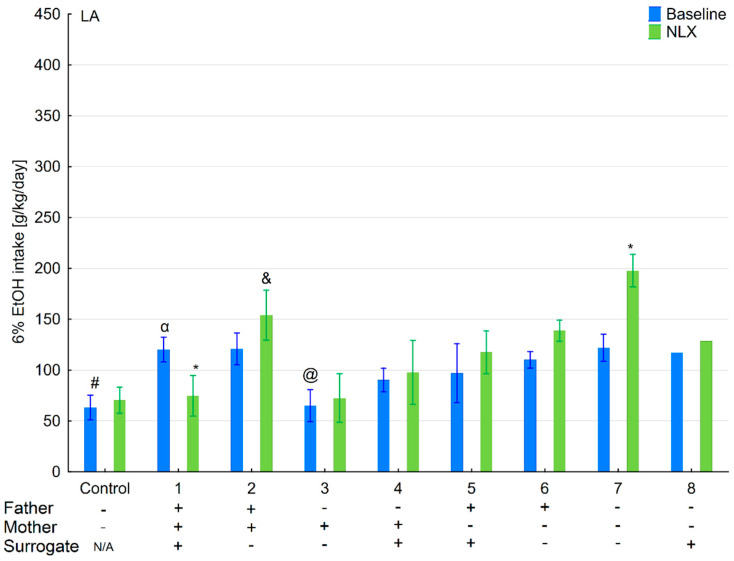
Effects of naloxone on 6% ethanol intake in the LA offspring reared by a HA surrogate. The LA offspring reared by their biological mothers were used as controls. Post hoc comparisons are marked when *p*-value is at least less than 0.05: *, baseline vs NLX; #, control baseline vs. 1,2,6,7,8 baseline; α, 1 baseline vs. 3 baseline; &, 2 baseline vs. 3 baseline; @, 3 baseline vs. 7,8 baseline.

**Figure 10:**
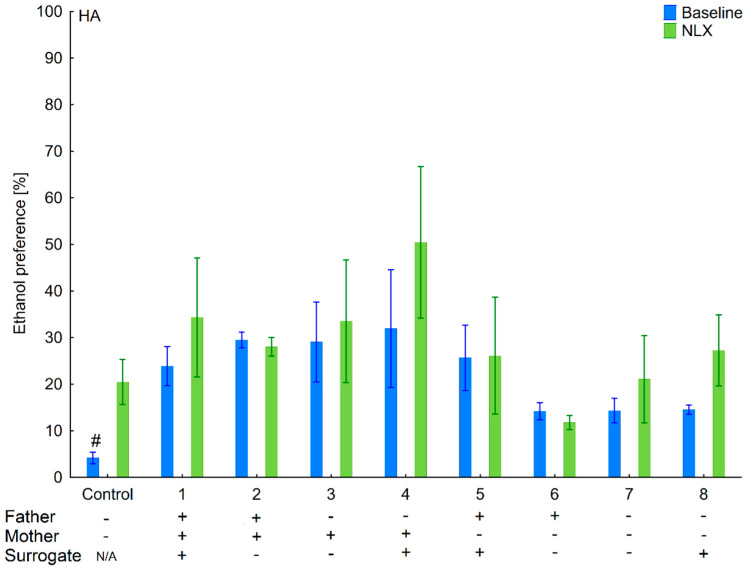
Effects of naloxone on 6% ethanol preference in the HA offspring reared by an LA surrogate. The HA offspring reared by their biological mothers were used as controls. Post hoc comparisons are marked when *p*-value is at least less than 0.05: #, control baseline vs. 2,3,4,5 baseline.

**Figure 11:**
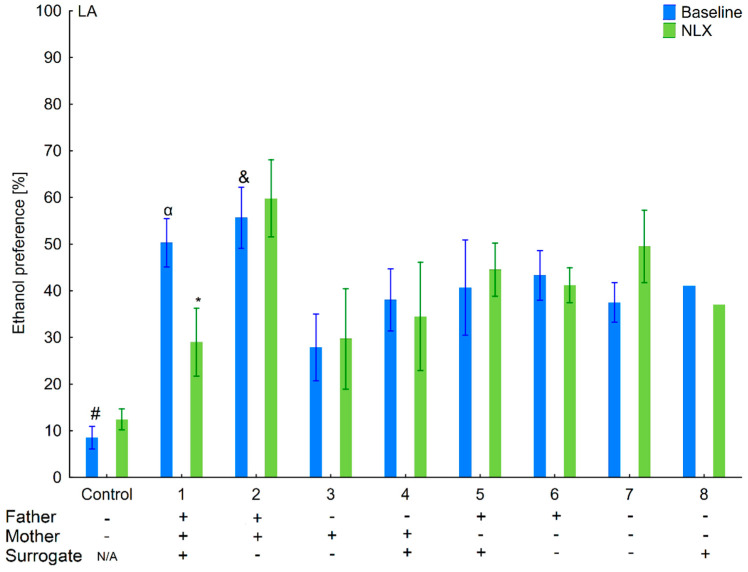
Effects of naloxone on 6% ethanol preference in the LA offspring reared by a HA surrogate. The HA offspring reared by their biological mothers were used as controls. Post hoc comparisons are marked when *p*-value is at least less than 0.05: *, baseline vs NLX; #, control baseline vs. 1,2,3,4,5,6,7 baseline; α, 1 baseline vs. 3 baseline; &, 2 baseline vs. 3 baseline.

Ethanol intake and preference were measured in the two-bottle free-choice paradigm in both HA and LA mice (Figure 8, Figure 9, Figure 10 and Figure 11). Two-way ANOVA taking line and variant as independent factors revealed that ethanol intake was different between lines (*F*(1,66) = 14.61; *p* = 0.001–line) but not variants (*F*(8,66) = 1.26; *p* = 0.28–variant). The interaction between line and variant failed to achieve statistical significance (*F*(8,66) = 2.07; *p* = 0.051), suggesting that ethanol intake was not influenced by adoption variant among lines, although a robust trend existed. However, ethanol preference was higher in the HA line (*F*(1,66) = 32.02; *p* < 0.001-line) as well as in some adoption variants (*F*(8,66) = 6.89; *p* < 0.001–variants). Moreover, effects of adoption on ethanol preference were similar in both lines as shown by an insignificant Line × Variant interaction (*F*(8,66) = 1.87; *p* = 0.08).

Further one-way ANOVA (variant as an independent factor) within lines showed that variant of adoption did not have a significant impact on further ethanol intake (*F*(8,35) = 1.36; *p* = 0.25–HA line; *F*(8,31) = 2.22; *p* = 0.053–LA line). Surprisingly, ethanol preference was affected by variant of adoption in both lines (*F*(8,35) = 3.14; *p* < 0.01–HA line; *F*(8,31) = 5.31; *p* < 0.001–LA line).

### 3.7. Effects of Naloxone on Ethanol Consumption and Preference in the HA and LA Mice Reared by Surrogates of the Opposite Line

Three-way ANOVA taking line, treatment, and variant as independent factors surprisingly indicated that administration of NLX did not affect ethanol intake (*F*(1,132) = 2.3; *p* = 0.13–treatment) (Figure 8, Figure 9, Figure 10 and Figure 11). Despite a non-significant treatment effect, the interaction between Line × Treatment (*F*(1,132) = 10.68; *p* < 0.01) was significant, showing that the effect of NLX administration on ethanol intake was more prominent in HA individuals. Moreover, a three-way ANOVA revealed that the treatment was more effective in some adoption variants (*F*(8,132) = 6.17; *p* < 0.001–Variant × Treatment interaction) concerning their ethanol intake. A significant Line × Variant × Treatment interaction (*F*(8,132) = 4.76; *p* < 0.001) pointed out that several adoption variants within the HA line were more sensitive to NLX treatment than in the LA line considering their ethanol intake.

Further two-way ANOVA (treatment and variant as independent factors) showed that NLX was effective in modifying ethanol intake in HA (*F*(1,70) = 10.64; *p* < 0.01–treatment) but not LA (*F*(1,62) = 1.99; *p* = 0.16–treatment) mice. In the HA line, we observed that administration of NLX in several variants had higher effect on ethanol intake than in others, as shown by a significant Variant × Treatment interaction (*F*(8,70) = 7.81 *p* < 0.001–HA line), which was not observed in LA mice (*F*(8,62) = 1.09; *p* = 0.38–LA line).

Three-way ANOVA (line, treatment and variant as independent factors) indicated that NLX was ineffective in altering ethanol preference (*F*(1,132) = 1.51, *p* = 0.22–treatment). Non-significant interactions showed that effect of NLX administration on ethanol preference was comparable between variants (*F*(8,132) = 0.50, *p* = 0.50–Variant × Treatment) and lines (*F*(1,132) = 2.07; *p* = 0.15–Line × Treatment). Additionally, a non-significant Line × Variant × Treatment interaction (*F*(8,132) = 0.77; *p* = 0.63) revealed that the effect of naloxone treatment on ethanol preference was comparable among variants within lines.

Analysis within the lines (treatment and variant as independent factors) revealed no effects of NLX on ethanol preference (*F*(1,70) = 3.87; *p* = 0.06–treatment in the HA line; *F*(1,62) = 0.02; *p* = 0.88–treatment in the LA line). The adoption variant did not alter the effect of NLX on ethanol preference, as shown by a non-significant Variant × Treatment interaction (*F*(8,70) = 0.47; *p* = 0.87–HA line; *F*(8,62) = 0.88; *p* = 0.54–LA line).

### 3.8. Changes in the Level of Depressive-Like Behaviors of the HA and LA Mice Reared by a Surrogate of the Opposite Line

**Figure 12:**
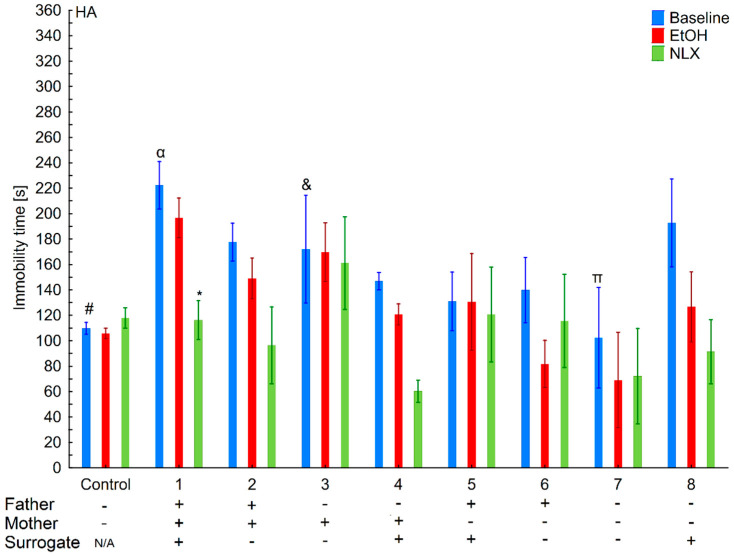
Effects of cross-fostering between the lines, ethanol consumption, and NLX treatment on depressive-like behaviors of the HA individuals reared by LA surrogate. The HA offspring reared by their biological mothers were used as the controls. Post hoc comparisons are marked when *p*-value is at least less than 0.05: *, EtOH vs. NLX; #, control baseline vs. 1 baseline; α, 1 baseline vs. 5,6,7 baseline; &, 2 baseline vs. 7 baseline; π–7 baseline vs. 8 baseline.

**Figure 13:**
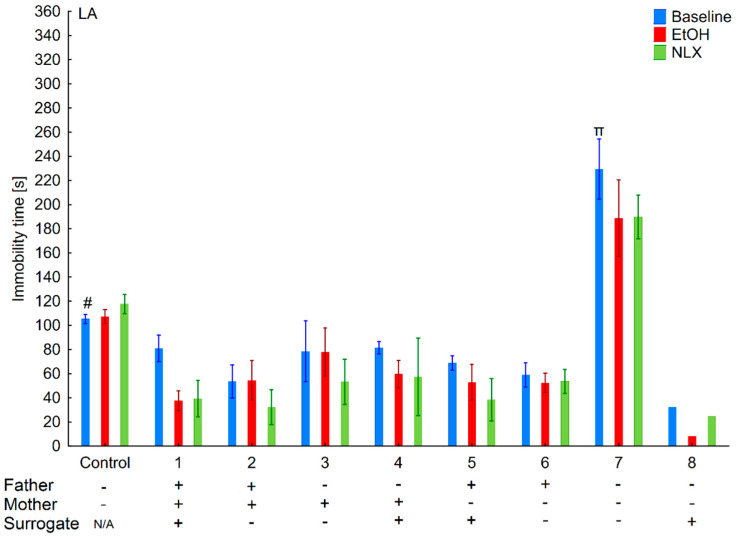
Effects of cross-fostering within the line, ethanol consumption, and NLX treatment on depressive-like behaviors of the LA individuals reared by HA surrogate. The LA offspring reared by their biological mothers were used as the controls. Post hoc comparisons are marked when *p*-value is at least less than 0.05: #, control baseline vs. 2,7 baseline; π–7 baseline vs. 1,2,3,4,5,6,7,8 baseline.

Two-way ANOVA (line and variant as independent factors) showed that HA mice remained immobile for a longer time than LA mice (*F*(1,64) = 32.66; *p* < 0.001–line) (Figure 12 and Figure 13). Moreover, depressive-like behaviors were more pronounced in mice from adoption variants within the HA line as confirmed by a significant Line × Variant interaction (*F*(8,64) = 6.88; *p* < 0.001).

Analysis within lines (variant as independent factor) revealed that variant of adoption had a significant effect only in LA (*F*(8,30) = 12.62; *p* < 0.001) but not in HA (*F*(8,34) = 2.21; *p* = 0.051) mice.

### 3.9. Effects of Ethanol Consumption on Depressive-Like Behaviors in the HA and LA Mice Reared by a Surrogate of the Same Line

Three-way ANOVA (line, EtOH intake and variant as independent factors) revealed that ethanol consumption had an anti-depressive effect (*F*(1,125) = 7.36; *p* < 0.01–EtOH intake) (Figure 12 and Figure 13). However, the effect of ethanol intake on depressive-like behaviors was comparable in the HA and LA mice, as revealed by a non-significant Line × Treatment interaction (*F*(1,125) = 0.42; *p* = 0.52). A non-significant Treatment × Variant (*F*(8,125) = 0.46; *p* = 0.88) interaction and Line × Treatment × Variant (*F*(8,125) = 0.29; *p* = 0.97) interaction pointed out that ethanol’s effect between adoption variants among lines was comparable.

Two-way ANOVA within the lines (EtOH intake and variant as independent factors) revealed that ethanol consumption reduced depressive-like behaviors more effectively in HA (*F*(1,67) = 4.79; *p* < 0.05–EtOH intake) than LA (*F*(1,58) = 3.61; *p* = 0.06–EtOH intake) mice. A non-significant Variant × Treatment interaction (*F*(8,67) = 0.38; *p* = 0.93–HA line; *F*(8,58) = 0.60; *p* = 0.77–LA line) indicated that adoption variant did not alter response to ethanol intake.

### 3.10. Effects of Naloxone on Depression-Like Behaviors in the La and Ha Mice Rearing by A Surrogate of the Opposite Line

Three-way ANOVA (line, variant, NLX treatment as independent factors) indicated that NLX had no effect on depressive-like behavior (*F*(1,122) = 2.24; *p* = 0.13–NLX treatment) (Figure 12 and Figure 13). Further analysis within the lines (variant and NLX treatment as independent factors) confirmed that depressive-like behaviors remained the same as before NLX treatment (*F*(1,66) = 2.80; *p* = 0.10–HA line; *F*(1,56) = 0.15; *p* = 0.70–LA line).

## 4. Discussion

A prominence of parental rearing on the offspring phenotype has been explored previously using a cross-fostering paradigm. Our study demonstrated that the offspring replacement, even without additional incentives such as ethanol, resulted in phenotypic changes in pups later in life. The observation is consistent with several previous studies based on comparable assumptions or similar methodologies. It has been documented that pups born by a C57BL/6 mouse and nursed by a BALB/cJ mothers demonstrate BALB-like behavioral traits [22]. In another study, maternal care was linked with changes in body weight and height of the pups divergently selected for these traits. Although the genotype influence prevailed [23,24], it was confirmed later on two distinct murine populations without any preselection considering weight or height criteria [25].

Additional inquiries showed a dominance of postnatal maternal care on the body mass changes in the offspring over prenatal influence, likely associated with breastfeeding [26]. Rearing by a surrogate may contribute to metabolic changes and, consequently, to hypertension or glucose intolerance [27]. However, in our study, we did not observe changes in body weight among offspring in different groups [data not shown]. Another feature analyzed about parental rearing is susceptibility to stress. Mice with low tolerance to hostile conditions but nursed by a resilient surrogate demonstrated decreased corticosterone levels in adulthood [28]. The importance of a surrogate effect on changes in behavioral and physiological responses to stress was further confirmed in rats that were particularly vulnerable to maternal care quality for the first week of life [29].

Additional evidence for rearing impact on phenotype is provided by a study in apomorphine susceptible (APO-SUS) and apomorphine unsusceptible (APO-UNSUS) rats. The APO-SUS rats display schizophrenic-like traits as a specific behavioral response to new objects in their environment. In contrast, the APO-UNSUS rats exhibit a normal repertoire of reactions to occurring changes. The offspring cross-fostering in this study had beneficial effects on phenotype in mature individuals as did behavioral normalization in APO-SUS and decline of locomotor activity in APO-UNSUS [30]. Although a strong relationship between parental rearing and progeny performance has been well-documented, our study showed no surrogate nursing effect on ethanol consumption and preference in the foster pups. There was no significant change in these parameters in mice having ethanol-naïve parents and ethanol-naïve surrogate. Our results seem to contradict the data collected from a previous study on an influence of cross-fostering of mice selected for high and low ethanol preference (HAP2, high alcohol-preferring, and LAP2, low alcohol-preferring). In this experiment, a significant decrease in ethanol consumption and preference was observed in HAP2 mice reared by a LAP2 surrogate, whereas there was no impact on LAP2 mice that had a HAP2 foster mother [31]. Data from research on human populations strongly suggest that genetic factors are mainly responsible for addiction vulnerability [31]. Children in foster families are more prone to alcohol addiction or illicit substance use when their birth parents had a history of alcohol or drug use disorder. In most such cases, handling by non-addicted foster families is insufficient to neutralize the genetic burden [32].

Alcohol misuse in foster parents and its effect on non-biological descendants is a separate issue. According to the 1992 National Longitudinal Alcohol Epidemiologic Survey, the risk of excessive and habitual alcohol consumption increases when both biological and foster parents misuse alcohol. The risk of becoming a heavy drinker increases by 5-fold in biological parents’ progeny with recognized alcohol use disorders (AUD) and 9-fold when both birth and foster parents are dependent on alcohol [33]. In our study, the HA offspring demonstrated increased ethanol consumption when only biological parents, not a surrogate, had access to it. Thus, ethanol habits presented by genetic parents decide predominantly on a later behavioral pattern copying in the offspring. The vast majority of research was conducted mainly among human populations in a form of observational studies.

A Prospective Collaborative Study on the Genetics of Alcoholism (COGA) indicated a higher risk of AUD in individuals whose biological parents were also afflicted. A child initiates alcohol consumption early in life and develops AUD, particularly if both parents are heavy drinkers or only the mother is [34]. Our experiments demonstrated that ethanol consumption exclusively by a biological mother did not affect ethanol intake to the same extent in the offspring. On the other hand, drinking ethanol by a biological father was associated with increased ethanol consumption by the HA offspring and decreased ethanol intake in LA mice. This specific phenomenon of the impact of a father with ethanol experience on ethanol consumption by foster pups may be explained by reduced activity of the endogenous opioid system in LA mice, eliciting an elevated urge to drink [20]. A lack of maternal effect might arise from the general dysfunction of the opioid system that affects mainly females [35]. Moreover, a study performed on CD1 mice proved that paternal ethanol exposure affects brain-derived neurotrophic factor (BDNF) and nerve growth factor (NGF) in their progeny, which may also be linked with higher ethanol preference in the ethanol-experienced father’s progeny [36]. Furthermore, a series of experiments indicated that paternal exposure to ethanol might decrease ethanol intake in progeny [37,38,39]. However, male offspring were more prone to anxiogenic effects of ethanol intake, which may explain the reason for lower doses consumed by them [38]. Another cohort study also revealed a link between AUD in both parents or only mothers and an increased risk of AUD onset in children, particularly females [40]. A sex-dependent consumption and preference in our model appeared to be without prominence as was tested in previous experiments; therefore, this factor could be ignored in present settings. The relevance of parental alcohol dependence in exacerbating the risk for early tobacco or marijuana use by their children has been confirmed as well [41,42].

Another trait we focused on in this study was the effect of rearing on depression. Epidemiological data indicate that genetic makeup is a major risk factor for depression in 40–50% of all cases [43]. Particularly, parental depression is strongly associated with mental disorders in their children. Anxiety, depression, and substance use disorders occur 3-fold more frequently in progeny from similarly burdened families [44], with the prevailing influence of both parents affected or only the mothers but not the fathers. This notion is also relevant in the foster offspring’s cases [45], likely because of stress inseparably associated with adoption. This observation was further verified in a research paper studying a genetic model of depression involving two lines of rats of distinct susceptibility to swim stress: a more depressive Flinder Sensitive Line (FSL) remaining more time immobile in the forced swim test than control Sprague Dawley (SD) rats. Cross-fostering between these two lines had more adverse effects on depressive-like symptoms in the SD offspring than in the FSL pups [46].

In our model, HA mice, being more prone to develop depression, had a higher immobility score after replacing the biological mother with a foster one. In contrast, LA mice spent less time without any movement. Similarly, C57BL/6 murine pups demonstrated more depressive-like symptoms following the original mother’s replacement with a surrogate one [47]. Late adoption (between 5 and 12 days of life) in rats has the same effect as early separation from a mother and promoted anxiety and depression-like behavior. Insufficient nursing by a foster dam may lead to epigenetic changes reflected by prolonged corticosterone release under stressful conditions, which presumably is a reason for the observed behavior pattern [48]. Similar to our results, a recent study performed on mice showed that paternal ethanol exposure does not lead to depressive-like behaviors in male progeny [49]. Interestingly, the same study reports that female offspring of ethanol-exposed fathers develop anxiety-like and depressive-like behaviors [49]. These findings suggest that ethanol-induced depression and anxiety in the progeny are strongly sex-dependent.

Next, we examined the impact of parental ethanol consumption on the development of depression in the offspring. HA mice had more pronounced depressive-like behaviors when biological and foster parents were ethanol users. Simultaneously, when only foster mothers were drinking ethanol and biological parents never used ethanol, we observed the disappearance of signs relevant to adverse mood. In contrast, LA mice, reared by surrogates consuming alcohol, became affected by depression. Clinical studies report more depressive syndromes and more frequent diagnoses of depression in adults with parents diagnosed with AUD (ACOAs–Adult Children of Alcoholics) than non-ACOAs [50]. It has been emphasized that parental substance misuse itself is not harmful to children, but rather exposure to the risk of experiencing undesirable, sometimes traumatic consequences associated indirectly with caregiver’s AUD [51]. According to a previous study, AUD, in some cases, may decrease parental attention and engagement in rearing, which leads to an underdeveloped ability to manage negative affective states in recipients of poor care. Moreover, emotional immaturity was frequently observed in offspring when the mother or father was using alcohol in excess. Unfortunately, the relationship of children with healthy parents did not compensate for the influence of AUD parents, and they usually develop depression when reaching adulthood [52]. Consistent results were obtained in similar studies on nicotine or cannabinoids dependence [53]. Our data confirmed the existing connection between ethanol consumption and depression.

Genetic and environmental factors mediate the development of these disorders. Major depressive disorder (MDD) in biological or foster parents increases susceptibility to developing mental problems in their offspring. In contrast, the risk of substance use disorders (SUD) in progeny elevates only when biological parents are affected, suggesting that environmental factors are more prevalent in the development of depression than addiction. Therefore, AUD leads to mental disorders such as depression through a negative impact on familial and social life [54]. Moreover, alcohol exposure elicits metabolic dysfunctions, i.e., a decrease in a methylenetetrahydrofolate reductase, a key enzyme in producing the active form of folic acid known to prevent depression development, which prompts a conclusion that alcohol may pave the way for mentally unstable status [55].

Another hypothesis is that depression promotes alcohol self-administration and possibly AUD, supported by known properties of alcohol as a stress suppressor and positive sensation inducer. Not so uncommon is alcohol misuse by subjects with depression to remove unpleasant symptoms, leading to dependence [56]. In our experimental model, ethanol had a modest anti-depressive effect on CMS in HA mice. Intrinsically resistant to CMS conditions, LA mice do not exhibit any changes in depressive-like behavior following ethanol intake [12]. It can be suggested that ethanol consumption by the biological father or a surrogate of the opposite line eliminates ethanol’s anti-depressive effect in the HA offspring. Interestingly, LA offspring of ethanol-naïve parents but reared by a foster mother exposed to ethanol demonstrated increased depressive-like behaviors. In this setting, ethanol had anti-depressive effects in LA offspring with biological parents and the foster mother drinking ethanol.

A novel aspect of our study was determining changes in naloxone’s effect on ethanol consumption and depression arisen following cross-fostering. Intraperitoneal injection of naloxone triggers a robust increase in ethanol consumption and preference in HA but not in LA mice. HA mice developed depressive-like behaviors following naloxone treatment, explaining the significant increase in ethanol intake [20]. Following HA mice cross-fostering, the influence of naloxone on ethanol consumption and preference was reduced, except for the variant with only the father allowed to drink ethanol that sustained naloxone-induced increase in ethanol consumption. A possible explanation of the observation is a reduction of naloxone’s effect on the above parameters in HA mice because of ethanol intake by a biological mother or a surrogate (similar results were obtained in a variant of nonbiological or foster parent drinking ethanol). Interestingly, despite increased ethanol consumption, naloxone limited depressive-like behavior in the offspring of the only ethanol-drinking father. Naloxone also revealed anti-depressive properties in mice of both biological parents having access to ethanol. Depressive-like behaviors of animals with a hypoactive opioid system, i.e., LA mice, in any cross-fostering study variant, remained intact despite naloxone administration. A comparable naloxone-mediated outcome was recognized in a previous study evaluating swim stress-induced analgesia in HA mice reared by LA surrogate, where naloxone showed reduced effects on SSIA level, probably as a result of a decrease in MOR receptors density following cross-fostering.

In the presented study, we used a complex approach for investigating the undertaken problem, which also resulted in the main limitation of our study, i.e., a relatively low sample size per group (*n* = 4–6). However, our previous study showed that this number is minimal sample size for obtaining reliable results from behavioral experiments [57]. A low number of individuals per group also results in a specific statistical approach, which considers parental variant as one, without dividing it into three separate factors–one per parent. The low sample size may cause information loss; however, we tried our best to create all possible parental variants to make it possible to show which parent had an impact on the parameters examined. The lack of ethanol intake measurement in parental generation can be considered a limitation of our study due to possible differences in consumption between lines. However, parental generation had access only to ethanol; we assumed that the intake of a 6% solution would be similar to physiological water consumption since ethanol was the only liquid available for them. From previous studies on HA and LA mice, we know that in time there is no increase of ethanol intake resulting from dependence development or tolerance to pleasurable effects of ethanol [11]. Moreover, a 6% ethanol solution does not produce aversive effects, which also justifies our assumption. As reported in different studies [58,59], another limitation of the presented experiment may be relatively low control of litter effect, since as mentioned above we used offspring from only two different litters. However, based on our previous reports, we know that ethanol intake and preference are similar across litters within HA and LA lines [11,12,20]. We believe that despite the many limitations of the current study, we provide many promising results that can be the basis for further experiments.

To summarize, based on our research findings derived from HA and LA progeny that were challenged by the interplay of several inherent and environmental factors—such as the genetic predisposition to depression, different ethanol use background of biological or surrogate parents, rearing by a foster caregiver, and treatment with an opioid antagonist—we have demonstrated that some external influences experienced postnatally, such as susceptibility to AUD, can be transmitted epigenetically from parents to next generations.

## 5. Conclusions

In this study, we showed that ethanol use by biological parents significantly alters ethanol intake and depressive-like behaviors in their offspring, indicating that genetics and epigenetic factors play a critical role in developing ethanol dependence and depression. In the presented research, ethanol consumption by surrogate mothers had an impact on depressive-like behavior. Still, it surprisingly did not alter ethanol intake in the foster children, which led us to conclude that environmental factors have a greater impact on the development of depression than ethanol dependence.

## Figures and Tables

**Table 1:** Experimental groups representation of HA or LA offspring used in the current study. +, free access only to ethanol; –, free access only to water.

Experimental Group	Parental Variant		
	Biological Father	Biological Mother	Surrogate Mother
1	**+**	**+**	**+**
2	**+**	**+**	**−**
3	**−**	**+**	**−**
4	**−**	**+**	**+**
5	**+**	**−**	**+**
6	**+**	**−**	**−**
7	**−**	**−**	**−**
8	**−**	**−**	**+**
Control	**−**	**−**	No transfer

**Table 2:** Number of animals in each experimental group.

Experimental Group	HA Within	HA Between	LA Witihn	LA Between
**1**	6♂	5♂	6♂	5♂
**2**	6♂	5♂	6♂	4♂
**3**	6♂	5♂	6♂	4♂
**4**	6♂	4♂	6♂	4♂
**5**	6♂	5♂	6♂	5♂
**6**	6♂	5♂	6♂	4♂
**7**	6♂	5♂	6♂	4♂
**8**	6♂	2♂ 2♀	6♂	4♂
**Control**	6♂	6♂	6♂	6♂

## Data Availability

The authors declare that the data supporting the finding of this study are available within the paper and its supplementary information files.

## Supplementary Materials

The following are available online at https://www.mdpi.com/article/10.3390/brainsci11050622/s1, Table S1: Significant comparisons in ethanol intake in the HA pups reared by HA surrogate.; Table S2: Significant comparisons in ethanol preference in the HA pups reared by HA surrogate.; Table S3: Significant comparisons in depressive-like behavior in the HA pups reared by HA surrogate.; Table S4: Significant comparisons in ethanol intake in the LA pups reared by LA surrogate.; Table S5: Significant comparisons in ethanol preference in the LA pups reared by LA surrogate.; Table S6: Significant comparisons in depressive-like behavior in the LA pups reared by LA surrogate.; Table S7: Significant comparisons in ethanol intake in the HA pups reared by LA surrogate.; Table S8: Significant comparisons in ethanol preference in the HA pups reared by LA surrogate.; Table S9: Significant comparisons in depressive-like behavior in the HA pups reared by LA surrogate.; Table S10: Significant comparisons in ethanol intake in the LA pups reared by HA surrogate.; Table S11: Significant comparisons in ethanol preference in the LA pups reared by HA surrogate.; Table S12: Significant comparisons in ethanol intake in the LA pups reared by HA surrogate.
