# Supplementary material for: The Influence of Cross-Fostering on Alcohol Consumption and Depressive-Like Behaviors in HA and LA Mice: The Role of the Endogenous Opioid System
Source: Brain Sci. 2021 May 13;11(5):622. doi: 10.3390/brainsci11050622 (PMC8152237; doi:10.3390/brainsci11050622)
Supplement: Supplementary file 1 [file brainsci-11-00622-s001.zip › brainsci-1180853-supplementary.pdf]

Table S1. Significant comparisons in ethanol intake in the HA pups reared by HA surrogate.

Ethanol intake within HA line			
**Group 1 vs Group 2**		**p-value**	**Direction in relation to group 1**
**Control baseline**	2 baseline	0,006	↑
**Control baseline**	6 baseline	0,011	↑
**Control baseline**	7 baseline	0,029	↑
**1 baseline**	2 baseline	<0,001	↑
**1 baseline**	4 baseline	0,012	↑
**1 baseline**	6 baseline	<0,001	↑
**1 baseline**	7 baseline	<0,001	↑
**2 baseline**	3 baseline	<0,001	↓
**2 baseline**	5 baseline	0,009	↓
**2 baseline**	8 baseline	0,007	↓
**3 baseline**	4 baseline	0,029	↑
**3 baseline**	6 baseline	<0,001	↑
**3 baseline**	7 baseline	<0,001	↑
**5 baseline**	6 baseline	0,017	↑
**5 baseline**	7 baseline	0,041	↑
**6 baseline**	8 baseline	0,015	↓
**7 baseline**	8 baseline	0,034	↓
**Control baseline**	Control NLX	<0,001	↑

Table S2. Significant comparisons in ethanol preference in the HA pups reared by HA surrogate.

Ethanol preference within HA line			
**Group 1 vs Group 2**		**p-value**	**Direction in relation to group 1**
**Control baseline**	2 baseline	<0,001	↑
**Control baseline**	4 baseline	<0,001	↑
**Control baseline**	5 baseline	0,008	↑
**Control baseline**	6 baseline	<0,001	↑
**Control baseline**	7 baseline	<0,001	↑
**Control baseline**	8 baseline	0,002	↑
**1 baseline**	2 baseline	<0,001	↑
**1 baseline**	4 baseline	0,008	↑
**1 baseline**	6 baseline	<0,001	↑
**1 baseline**	7 baseline	0,001	↑
**2 baseline**	3 baseline	<0,001	↓
**2 baseline**	5 baseline	0,001	↓
**2 baseline**	8 baseline	0,004	↓
**3 baseline**	4 baseline	0,01	↑
**3 baseline**	6 baseline	<0,001	↑
**3 baseline**	7 baseline	0,002	↑
**5 baseline**	6 baseline	<0,001	↑
**5 baseline**	7 baseline	<0,001	↑
**6 baseline**	8 baseline	0,002	↓
**2 baseline**	2 NLX	0,035	↑
**3 baseline**	3 NLX	0,029	↑
**4 baseline**	4 NLX	0,027	↑
**5 baseline**	5 NLX	<0,001	↑
**8 baseline**	8 NLX	<0,001	↑

Table S3. Significant comparisons in depressive-like behavior in the HA pups reared by HA surrogate.

Depressive-like behavior within HA line			
**Group 1 vs Group 2**		**p-value**	**Direction in relation to group 1**
**1 baseline**	3 baseline	<0,001	↓
**1 baseline**	4 baseline	<0,001	↓
**1 baseline**	5 baseline	0,004	↓
**1 baseline**	7 baseline	0,046	↓
**1 baseline**	8 baseline	0,001	↓
**2 baseline**	3 baseline	0,014	↓
**2 baseline**	4 baseline	0,049	↓
**3 baseline**	6 baseline	0,046	↑
**2 baseline**	2 EtOH	0,006	↓
**7 baseline**	7 EtOH	0,032	↓

Table S4. Significant comparisons in ethanol intake in the LA pups reared by LA surrogate.

**Ethanol intake witihin LA line**			
**Group 1 vs Group 2**		**p-value**	**Direction in relation to group 1**
**Control baseline**	1 baseline	<0,001	↑
**Control baseline**	3 baseline	<0,001	↑
**Control baseline**	7 baseline	0,031	↑
**Control baseline**	8 baseline	<0,001	↑
**1 baseline**	2 baseline	<0,001	↓
**1 baseline**	4 baseline	<0,001	↓
**1 baseline**	5 baseline	<0,001	↓
**1 baseline**	6 baseline	<0,001	↓
**1 baseline**	7 baseline	0,002	↓
**2 baseline**	3 baseline	<0,001	↑
**2 baseline**	6 baseline	0,039	↓
**2 baseline**	8 baseline	0,017	↑
**3 baseline**	4 baseline	<0,001	↓
**3 baseline**	5 baseline	<0,001	↓
**3 baseline**	6 baseline	<0,001	↓
**3 baseline**	7 baseline	0,001	↓
**4 baseline**	8 baseline	0,001	↑
**5 baseline**	8 baseline	0,001	↑
**6 baseline**	7 baseline	0,003	↑
**6 baseline**	8 baseline	<0,001	↑

Table S5. Significant comparisons in ethanol preference in the LA pups reared by LA surrogate.

Ethanol preference within LA line			
**Group 1 vs Group 2**		**p-value**	**Direction in relation to group 1**
**Control baseline**	1 baseline	<0,001	↑
**Control baseline**	2 baseline	<0,001	↑
**Control baseline**	3 baseline	<0,001	↑
**Control baseline**	4 baseline	0,012	↑
**Control baseline**	5 baseline	<0,001	↑
**Control baseline**	6 baseline	0,018	↑
**Control baseline**	7 baseline	<0,001	↑
**Control baseline**	8 baseline	<0,001	↑
**2 baseline**	3 baseline	0,029	↑
**2 baseline**	4 baseline	0,009	↓
**2 baseline**	6 baseline	0,003	↓
**3 baseline**	4 baseline	<0,001	↓
**3 baseline**	5 baseline	0,001	↓
**3 baseline**	6 baseline	<0,001	↓
**3 baseline**	7 baseline	0,029	↓
**4 baseline**	7 baseline	<0.001	↑
**4 baseline**	8 baseline	<0.001	↑
**5 baseline**	8 baseline	0,044	↑
**6 baseline**	7 baseline	0,003	↑
**6 baseline**	8 baseline	<0,001	↑
**5 baseline**	5 NLX	0,012	↑
**6 baseline**	6 NLX	0,013	↑
**7 baseline**	7 NLX	0,011	↑

Table S6. Significant comparisons in depressive-like behavior in the LA pups reared by LA surrogate.

Depressive-like behavior within LA line			
**Group 1 vs Group 2**		**p-value**	**Direction in relation to group 1**
**Control baseline**	7 baseline	0,03	↓
**Control baseline**	8 baseline	0,019	↓
**1 baseline**	5 baseline	0,017	↑
**2 baseline**	5 baseline	0,012	↑
**5 baseline**	7 baseline	0,002	↓
**5 baseline**	8 baseline	<0,001	↓
**5 baseline**	5 EtOH	0,008	↓
**7 baseline**	7 EtOH	0,048	↓

Table S7. Significant comparisons in ethanol intake in the HA pups reared by LA surrogate.

Ethanol intake in the HA pups reared by LA mother			
**Group 1 vs Group 2**		**p-value**	**Direction in relation to group 1**
**2 baseline**	6 baseline	0,039	↓
**2 baseline**	7 baseline	0,041	↓
**Control baseline**	Control NLX	<0,001	↑

Table S8. Significant comparisons in ethanol preference in the HA pups reared by LA surrogate.

Ethanol preference in the HA pups reared by LA mother			
**Group 1 vs Group 2**		**p-value**	**Direction in relation to group 1**
**Control baseline**	2 baseline	0,006	↑
**Control baseline**	3 baseline	0,021	↑
**Control baseline**	4 baseline	0,016	↑
**Control baseline**	5 baseline	0,046	↑

Table S9. Significant comparisons in depressive-like behavior in the HA pups reared by LA surrogate.

Depressive-like behavior in the HA pups reared by LA mother			
**Group 1 vs Group 2**		**p-value**	**Direction in relation to group 1**
**Control baseline**	1 baseline	0,004	↑
**1 baseline**	5 baseline	0,013	↓
**1 baseline**	6 baseline	0,025	↓
**1 baseline**	7 baseline	0,001	↓
**2 baseline**	7 baseline	0,0498	↓
**7 baseline**	8 baseline	0,027	↑

Table S10. Significant comparisons in ethanol intake in the LA pups reared by HA surrogate.

Ethanol intake in the LA pups reared by HA mother			
**Group 1 vs Group 2**		**p-value**	**Direction in relation to group 1**
**Control baseline**	1 baseline	0,016	↑
**Control baseline**	2 baseline	0,006	↑
**Control baseline**	6 baseline	0,046	↑
**Control baseline**	7 baseline	0,023	↑
**Control baseline**	8 baseline	0,023	↑
**1 baseline**	3 baseline	0,023	↓
**2 baseline**	3 baseline	0,009	↓
**3 baseline**	7 baseline	0,032	↑
**3 baseline**	8 baseline	0,026	↑
**1 baseline**	1 NLX	0,007	↓

Table S11. Significant comparisons in ethanol preference in the LA pups reared by HA surrogate.

Ethanol preference in the LA pups reared by HA mother			
**Group 1 vs Group 2**		**p-value**	**Direction in relation to group 1**
**Control baseline**	1 baseline	<0,001	↑
**Control baseline**	2 baseline	<0,001	↑
**Control baseline**	3 baseline	0,039	↑
**Control baseline**	4 baseline	0,004	↑
**Control baseline**	5 baseline	<0,001	↑
**Control baseline**	6 baseline	<0,001	↑
**Control baseline**	7 baseline	0,004	↑
**1 baseline**	3 baseline	0,002	↓
**2 baseline**	3 baseline	0,005	↓
**1 baseline**	1 NLX	0,029	↓

Table S12. Significant comparisons in ethanol intake in the LA pups reared by HA surrogate.

Depressive-like behavior in the LA pups reared by HA mother			
**Group 1 vs Group 2**		**p-value**	**Direction in relation to group 1**
**Control baseline**	2 baseline	0,029	↓
**Control baseline**	7 baseline	<0,001	↑
**7 baseline**	1 baseline	<0,001	↓
**7 baseline**	2 baseline	<0,001	↓
**7 baseline**	3 baseline	<0,001	↓
**7 baseline**	4 baseline	<0,001	↓
**7 baseline**	5 baseline	<0,001	↓
**7 baseline**	6 baseline	<0,001	↓
**7 baseline**	7 baseline	<0,001	↓
**7 baseline**	8 baseline	<0,001	↓
